# Sex-Disparity in the Association Between Birthweight and Cardiovascular Parameters in 4-Year-Old Children: A Chinese Cohort Study

**DOI:** 10.3389/fnut.2021.756512

**Published:** 2021-10-26

**Authors:** Hualin Wang, Bowen Du, Yujian Wu, Zhuoyan Li, Yiwei Niu, Fengxiu Ouyang, Jian Wang, Sun Chen, Kun Sun

**Affiliations:** ^1^Department of Pediatric Cardiology, Xinhua Hospital, School of Medicine, Shanghai Jiao Tong University, Shanghai, China; ^2^Heart Center, Guangzhou Women and Children's Medical Center, Guangzhou, China; ^3^Ministry of Education, Shanghai Key Laboratory of Children's Environmental Health, Xinhua Hospital, School of Medicine, Shanghai Jiao Tong University, Shanghai, China

**Keywords:** sex-disparity, birthweight, cardiovascular parameters, left ventricular hypertrophy, birth cohort study

## Abstract

**Background:** Sex-related differences in cardiovascular parameters have been well documented in adults, and the impact of birthweight on cardiovascular health in later life has been acknowledged. However, data was limited regarding the association between birthweight and cardiovascular outcomes at an early age, and the sex-disparity in the association remained unclear.

**Objective:** To investigate the association between birthweight and cardiovascular parameters in 4-year-old children. Furthermore, to explore whether sex-disparity exist in this association or in cardiovascular risk.

**Methods:** Follow-up data from the Shanghai Birth Cohort (SBC) was analyzed. Detailed perinatal information including both maternal and offspring datum were recorded. Blood pressure, echocardiography, and anthropometry assessment were conducted during the follow-up of 4-year-old children. Linear regression models were used to analyze the association between birthweight and left ventricle (LV) structure and function changes in each sex and birthweight category. Multivariable logistic regression models were used to compare risk of left ventricular hypertrophy (LVH) in different birthweight subgroups.

**Results:** Overall, macrosomia was significantly associated with thickened LV posterior wall thickness in systole [LVPWs, (β = 0.26, 95% CI: 0.06, 0.45)] and diastole [LVPWd, (β = 0.18, 95% CI: 0.06, 0.30)], and thickened interventricular septal thickness in diastole [IVSd, (β = 0.16, 95% CI: 0.05, 0.28)]. Boys with macrosomia showed a higher left ventricle mass index [LVMI, (β = 1.29, 95% CI: 0.14, 2.43)], thickened LVPWs (β = 0.30, 95% CI: 0.05, 0.56) and LVPWd (β = 0.21, 95% CI: 0.06, 0.36), and thickened IVSd (β = 0.23, 95% CI: 0.09, 0.36). However, no significant association of structural changes was found in girls. Furthermore, an increased risk of LVH was found solely in macrosomic boys (OR = 2.79, 95% CI: 1.17, 6.63).

**Conclusion:** Children with macrosomia developed cardiovascular changes as early as 4 years of age. Macrosomia was associated with LV structural changes and higher LVH risk in pre-school-aged boys, while no association was found in girls.

## Introduction

The impact of birthweight on cardiovascular health in later life has been acknowledged (1, 2). Previous studies found that children diagnosed with low birthweight (LBW) or macrosomia were prone to develop increased risk of cardiovascular disease in later life (3–5). This phenomenon was in accordance with the Barker hypothesis (6), which proposed that adverse prenatal exposure could alter metabolism and physiology in later life, and lead to increased risk of adult cardiovascular disease (3, 4, 7).

Sex-disparity has been reported to exist in most non-communicable diseases and certain infectious diseases (8–10). There was sufficient evidence indicating the sex difference in the incidence and prognosis of cardiovascular diseases (11–13). Previous studies demonstrated that sex-disparity in cardiovascular parameters could begin in early adolescence (14), but data at an early age was limited. As far as we know, there has been no report on the sex difference in the association between cardiovascular health and birthweight in pre-school-aged children. Thus, the role of sex-disparity in the development of cardiovascular disease in early childhood remained unclear.

Based on the platform of Shanghai Birth Cohort (SBC), we aimed to examine the relationship between birthweight with the cardiovascular parameters, including blood pressure, and LV geometry and function, and further explore if sex-disparity existed during early childhood. The sex-specific risk of left ventricular hypertrophy (LVH) contributed by birthweight would also be investigated in this study.

## Materials and Methods

### Study Design and Participants

The SBC was an ongoing prospective cohort study conducted in four large tertiary-level hospitals in Shanghai, China. The protocol of SBC has been previously described in detail (15, 16). Volunteer couples were recruited during preconception care or early pregnancy period from 2013 to 2016. Face-to-face interviews were conducted by trained nurses to collect information including parental age, race, educational levels, occupation, family income, alcohol use, and smoking status during pregnancy. The data related to newborns, regarding sex, gestational age, and anthropometric parameters, were recorded at birth by trained nurses. Information on maternal blood glucose and blood pressure levels was also collected from hospital records with the consent of patients. Those families still residing in Shanghai were invited to the prospective study. Detailed follow-up was provided for children at the age of 2 years and then at 4 years. Miscarriage, stillbirth, children with congenital heart defects, loss to follow-up in 4-year-olds, and without birthweight data were excluded. A total of 1,193 mother–offspring pairs were included in this study. Written informed consent was obtained from participants, and the study was approved by the Ethical Committee of Xinhua Hospital affiliated to Shanghai Jiao Tong University School of Medicine (no. XHEC-C-2013-001-2).

### Anthropometric Measurements

A complete anthropometric evaluation was conducted for all children at birth, the age of 2 years, and 4 years according to the standardized protocol. Height was measured barefoot twice to the nearest 0.1 cm. Weight was measured with light clothing and no shoes to the nearest 0.1 kg. The average of two repeats was collected, and the third measurement was conducted if the two readings differed by 0.1 kg or 0.1 cm. Body mass index (BMI) was calculated as weight (kg)/height^2^ (m^2^).

The classification of birthweight was conducted according to the World Health Organization (17). Macrosomia was defined as a birthweight over 4,000 g. Normal birthweight (NBW) was defined as a birthweight between 2,500 and 4,000 g, and low birthweight (LBW) was <2,500 g.

### Measurement of Blood Pressure

Blood pressure was measured from the left arm with appropriately sized cuff according to the guideline set by the American Academy of Pediatrics in 2017 (18), using the professional automatic BP monitor OMRAN HBP-1300 (Omron Healthcare, Guangzhou, CHINA) (19). Blood pressure was measured three times with a 5 min gap between replicates, after a comfortable rest of 5 min. Measurements were done by trained coordinators in duplicate and averaged.

### Measurement of Cardiovascular Parameters in 4-Year-Olds

Transthoracic echocardiography was performed for all children at the age of 4 years, by two experienced echocardiographers using an EPIQ 7C ultrasound system (Philips Healthcare, Andover, USA) equipped with S8-3 (8–3 MHz) and X5-1 (1–5 MHz) probes. Standard techniques were used to obtain M-mode, 2D, and Doppler measurements in accordance with the American Society of Echocardiography (20, 21). Quantitative measurements were obtained offline by well-trained investigators blinded to the data of subjects.

The dimensions of left ventricle (LV) were obtained from M-mode at parasternal short-axis view, including LV internal diameter indiastole and systole (LVDd and LVDs), interventricular septum thickness in diastole and systole (IVSd and IVSs), LV posterior wall thicknesses in diastole and systole (LVPWd and LVPWs), and left ventricular ejection fraction (LVEF) and fractional shortening (LVFS). Relative wall thickness (RWT) was calculated as (LVPWd + IVSd)/LVDd (22). The LV mass (LVM) was determined by the anatomically validated formula of Devereux (23) and the LVM index (LVMI) was calculated by dividing the LVM by the height^2.7^ (24). The sex-specific 95th percentiles of LVMI derived from the cohort were used as the cutoff points since there was no recommended standard on LVMI for 4-year-old children in China (25). LVMI = 33.24 g/m^2.7^ in girls and LVMI = 33.76 g/m^2.7^ in boys represented the sex-specific 95th percentiles in the cohort. The LVH was defined as an LVMI more than the sex-specific 95th percentiles.

The ratio of early diastolic flow velocity (E) and late diastolic flow velocity (a) across mitral valve (E/a) was obtained by pulse wave Doppler in the apical 4-chamber view. We also got the left ventricle ejection time (ET), isovolumic contraction time (ICT), and isovolumic relaxation time (IRT) in the apical 5-chamber view with the aid of pulse wave Doppler. The Tei index was calculated using the formula: Tei index = (ICT + IRT)/ET.

A 2-D speckle-tracking echocardiography (STE) analysis was performed using the commercial QLAB version 10.5 software (Philips Healthcare, Andover, USA) (26). To determine the global longitudinal strain (GLS), the software tracked the full wall region of interest automatically in the three apical views at the end of the diastole and allowed us to adjust the tracking when necessary. In this case, a 17-segment model was used.

A 2-D ultrasound examination of both common carotid arteries was also conducted using the same high-resolution EPIQ 7C ultrasound equipped with linear array transducer (L15-7io; Philips Healthcare, Andover, USA) following a standardized protocol. Carotid intima-media thickness (cIMT) was determined as the distance between the lumen–intima interface and the media–adventitia interface. The measurement was performed twice on each side and the mean value was used for final analysis.

### Statistical Analysis

Continuous variables were reported as means with standard deviation or means with minimum and maximum, and categorical variables were reported as percentages. One-way analysis of variance (one-way ANOVA) and Chi-square tests were used for comparison of basic characteristics and cardiovascular parameters among different birthweight groups in all children or different gender, and Bonferroni correction was used for multiple comparisons. Linear regression was used to assess all children and sex-specific associations between birthweight and cardiovascular parameters, using the NBW group as reference. Four models were conducted: model 1 was a crude model; model 2 was adjusted for maternal factors including maternal age, ethnicity (Han/Hui/Manchu/Mongolian/Zang/Uighur/Zhuang/Others/ unknown), educational level (never attended any school/primary school/junior high school/senior high school or technical school/junior college/undergraduate/postgraduate and above), alcohol intake (yes/no) and smoking status (yes/no) during pregnancy, gestational diabetes mellitus (yes/no), and hypertensive disorder during pregnancy (yes/no); model 3 was further adjusted for gestational age; model 4 was further adjusted for current BMI. Maternal age, gestational age, and current BMI were treated as continuous variables in the regression analyses. Due to the low incidence of LVH in LBW group, we only explored the risk of LVH in macrosomia group with NBW group as reference. Logistic regression models were used to estimate the risk of left ventricular hypertrophy (LVH) in different birthweight subgroups, and maternal factors, gestational age, current BMI, and current blood pressure, including SBP and DBP were adjusted. All analyses were performed using Stata 14.0 software (StataCorp, College Station, TX, USA) and were two-sided, with *p* < 0.05 considered as statistically significant.

## Results

### Association of Birthweight With Cardiovascular Outcome in 4-Year-Olds

A total of 616 boys and 577 girls were included in this study. Among all the 1,193 children, 46 children were diagnosed with LBW (3.86%), 963 children were diagnosed with NBW (80.72%), and 184 children were diagnosed with macrosomia (12.24%). Children showed LV structure changes at the age of 4 years, including LVPWs (*P* = 0.006), LVPWd (*P* = 0.004), LVDs (*P* = 0.017), and LVDd (*P* = 0.006), which were significantly different among three birthweight subgroups. However, the prevalence of LVH and other cardiovascular parameters, including blood pressure, LV structure parameters, and cIMT, showed no significant differences. The detailed information including demographic and anthropometric features of different birthweight subgroups is shown in Table 1.

**Table 1:** Basic and cardiovascular characteristics of different birthweight groups.

	**LBW**	**NBW**	**Macrosomia**	***P***
	**3.86% (*n* = 46)**	**80.72% (*n* = 963)**	**12.24% (*n* = 184)**	
Birthweight (g)	2187.2 ± 328.3	3313.6 ± 356.3	4237.0 ± 224.3	
**Gender**				
Boy, N (%)	23 (50.0%)	490 (50.8%)	103 (56.0%)	0.437
Girl, N (%)	23 (50.0%)	473 (49.2%)	81 (44.0%)	0.437
Gestational age (weeks)	35.95 (29.00, 40.40)	39.62 (33.14, 41.86)	39.82 (38.00, 41.43)	<0.001
Height at 4-yr (cm)	106.49 ± 4.31	107.07 ± 4.42	108.66 ± 4.93	<0.001
Weight at 4-yr (kg)	16.93 ± 2.09	17.13 ± 2.47	18.18 ± 3.18	<0.001
BMI at 4-yr (kg/m^2^)	14.50 ± 2.61	14.75 ± 2.16	15.23 ± 2.13	0.013
SBP (mmHg)	99.65 ± 8.12	97.87 ± 7.74	98.78 ± 7.67	0.148
DBP (mmHg)	58.62 ± 6.09	57.41 ± 6.09	57.31 ± 6.82	0.444
**LV structure at 4 years**				
LVMI (g/m^2.7^)	24.89 ± 4.00	26.17 ± 4.66	26.79 ± 5.28	0.079
LVPWs (mm)	7.78 ± 0.92	7.83 ± 0.90	8.08 ± 1.13	0.006
LVPWd (mm)	3.94 ± 0.54	4.14 ± 0.57	4.28 ± 0.72	0.004
LVDs (mm)	22.13 ± 1.84	22.52 ± 1.94	22.95 ± 2.10	0.017
LVDd (mm)	34.88 ± 2.55	35.40 ± 2.51	36.03 ± 2.66	0.006
IVSs (mm)	6.37 ± 0.88	6.56 ± 0.96	6.67 ± 1.14	0.215
IVSd (mm)	3.85 ± 0.65	3.79 ± 0.57	3.88 ± 0.66	0.156
RWT	0.23 ± 0.04	0.24 ± 0.04	0.24 ± 0.05	0.244
**LV function at 4 years**				
E/a	1.77 ± 0.34	1.80 ± 0.33	1.79 ± 0.38	0.873
Tei index	0.45 ± 0.06	0.42 ± 0.06	0.43 ± 0.07	0.337
LVEF (%)	60.08 ± 4.98	59.24 ± 4.59	59.16 ± 4.72	0.699
GLS (%)	23.33 ± 2.28	23.60 ± 2.28	23.59 ± 2.51	0.873
cIMT (mm)	41.26 ± 4.69	41.04 ± 4.56	40.92 ± 4.59	0.939
LVH, N (%)	1 (2.17%)	61 (6.33%)	19 (10.33%)	0.091

*M, male; F, female; LBW, low birthweight; NBW, normal birthweight; SBP, systolic blood pressure; DBP, diastolic blood pressure; BMI, body mass index; LV, left ventricle; LVMI, LV mass indexed to the height in m^2.7^; LVPWs, LV posterior wall thicknesses in systole; LVPWd, LV posterior wall thicknesses in diastole; LVDs, LV internal diameter in systole; LVDd, LV internal diameter in diastole; IVSs, interventricular septum thickness in systole; IVSd, interventricular septum thickness in diastole; RWT, relative wall thickness; E, mitral early wave velocities; a, mitral late wave velocities; LVEF, LV ejection fraction; GLS, global longitudinal strain; cIMT, carotid intima-media thickness; LVH, left ventricle hypertrophy. Values were expressed as means ± standard deviation or means with minimum and maximum. P-values were calculated using One-way ANOVA tests or Chi-square tests*.

**Figure 1:**
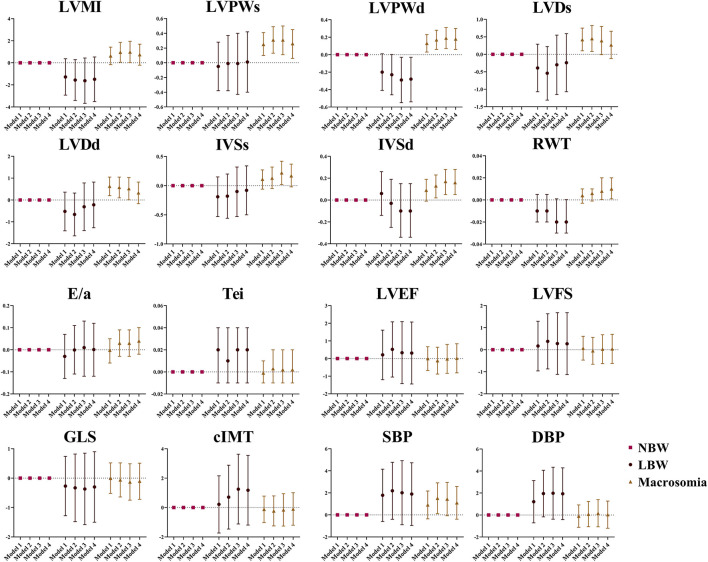
The association between birthweight and cardiovascular parameters at the age of 4 years. Data was presented as mean difference with error bars displaying 95% confidence intervals for multivariable linear regression compared to participants with normal birthweight. Model 1: crude model; Model 2: adjusted for maternal factors including maternal age, ethnicity, educational level, alcohol intake (yes/no) and smoking status (yes/no) during pregnancy, gestational diabetes mellitus (yes/no), and hypertensive disorder during pregnancy (yes/no); Model 3: further adjusted for gestational age; Model 4: further adjusted for current BMI.

The association between birthweight and cardiovascular parameters was further investigated among all the subjects. Positive association was found between children with macrosomia and LVPWs (β = 0.26, 95% CI: 0.06, 0.45), LVPWd (β = 0.18, 95% CI: 0.06, 0.30), IVSd (β = 0.16, 95% CI: 0.05, 0.28), and RWT (β = 0.01, 95% CI: 0.001, 0.02). Negative association was found between LBW and LVPWd (β = −0.28, 95% CI: −0.54, −0.03). These results are shown in Figure 1.

### Sex-Disparity in the Association of Birthweight With Cardiovascular Outcome in 4-Year-Olds

Among the children in this study, 23 (3.73%) were diagnosed with LBW and 103 (16.72%) were diagnosed with macrosomia among boys, while 23 (3.99%) were diagnosed with LBW and 81 (14.04%) were diagnosed with macrosomia among girls. Comparisons of cardiovascular parameters among different birthweight groups by gender are demonstrated in Table 2. The LV structure variables such as LVMI (*P* = 0.007), LVPWs (*P* = 0.008), LVPWd (*P* = 0.004), IVSd (*P* = 0.027), and the prevalence of LVH (*P* = 0.014) were significantly different among different birthweight groups in boys, which was not the case in girls. The blood pressure, LV function, and cIMT showed no significant difference in both boys and girls.

**Table 2:** Sex-specific basic and cardiovascular characteristics of different birthweight groups.

**Cardiac parameters**	**LBW**	**NBW**	**Macrosomia**	***P*-value**
**Boy**				
SBP at 4 years (mmHg)	101.39 ± 8.07	99.12 ± 7.92	99.76 ± 7.66	0.348
DBP at 4 years (mmHg)	56.95 ± 7.09	57.15 ± 6.29	57.04 ± 6.46	0.981
LVMI (g/m^2.7^)	24.75 ± 3.50	26.80 ± 4.25	28.06 ± 5.36^#^^§^	0.007
LVPWs (mm)	8.02 ± 0.85	7.95 ± 0.94	8.29 ± 1.13^#^	0.008
LVPWd (mm)	3.95 ± 0.52	4.21 ± 0.56	4.39 ± 0.73^#^^§^	0.004
LVDs (mm)	22.79 ± 2.06	22.94 ± 1.85	23.43 ± 2.06^#^	0.068
LVDd (mm)	36.16 ± 2.64	36.10 ± 2.40	36.76 ± 2.39^#^	0.052
IVSs (mm)	6.61 ± 0.90	6.77 ± 0.97	6.83 ± 1.04	0.666
IVSd (mm)	3.74 ± 0.37	3.80 ± 0.53	3.96 ± 0.61^#^	0.027
RWT	0.22 ± 0.03	0.24 ± 0.04	0.24 ± 0.05	0.115
E/a	1.69 ± 0.26	1.79 ± 0.33	1.81 ± 0.41	0.352
Tei index	0.44 ± 0.06	0.44 ± 0.07	0.43 ± 0.07	0.574
LVEF (%)	59.94 ± 3.87	58.87 ± 4.51	58.83 ± 4.16	0.731
GLS (%)	24.19 ± 1.93	23.44 ± 2.28	23.33 ± 2.42	0.520
cIMT (mm)	40.83 ± 4.22	41.33 ± 4.68	41.31 ± 4.34	0.946
LVH, N (%)	0 (0.00%)	32 (6.53%)	15 (14.56%) ^§^	0.014
**Girl**				
SBP at 4 years (mmHg)	97.83 ± 7.95	96.58 ± 7.37	97.32 ± 7.56	0.576
DBP at 4 years (mmHg)	60.38 ± 7.57	57.68 ± 5.90	57.63 ± 7.32	0.145
LVMI (g/m^2.7^)	25.01 ± 4.48	25.51 ± 4.98	25.12 ± 4.68	0.770
LVPWs (mm)	7.57 ± 0.94	7.70 ± 0.85	7.80 ± 1.07	0.535
LVPWd (mm)	3.93 ± 0.58	4.06 ± 0.57	4.11 ± 0.67	0.496
LVDs (mm)	21.59 ± 1.47	22.10 ± 1.92	22.28 ± 1.99	0.382
LVDd (mm)	33.81 ± 1.97	34.68 ± 2.41	35.04 ± 2.67	0.148
IVSs (mm)	6.18 ± 0.84	6.35 ± 0.90	6.46 ± 1.23	0.465
IVSd (mm)	3.94 ± 0.81	3.78 ± 0.61	3.78 ± 0.70	0.573
RWT	0.23 ± 0.04	0.24 ± 0.04	0.24 ± 0.05	0.952
E/a	1.81 ± 0.36	1.80 ± 0.32	1.78 ± 0.34	0.822
Tei index	0.45 ± 0.07	0.43 ± 0.06	0.43 ± 0.08	0.273
LVEF (%)	60.24 ± 6.20	59.65 ± 4.63	59.69 ± 5.53	0.930
GLS (%)	22.38 ± 2.33	23.76 ± 2.27	24.02 ± 2.64	0.138
cIMT (mm)	41.61 ± 5.20	40.75 ± 4.43	40.42 ± 4.91	0.704
LVH, N (%)	1 (3.40%)	29 (6.13%)	4 (4.94%)	0.842

*LBW, low birthweight; NBW, normal birthweight; SBP, systolic blood pressure; DBP, diastolic blood pressure; BMI, body mass index; LV, left ventricle; LVMI, LV mass indexed to the height in m^2.7^; LVPWs, LV posterior wall thicknesses in systole; LVPWd, LV posterior wall thicknesses in diastole; LVDs, LV internal diameter in systole; LVDd, LV internal diameter in diastole; IVSs, interventricular septum thickness in systole; IVSd, interventricular septum thickness in diastole; RWT, relative wall thickness; E, mitral early wave velocities; a, mitral late wave velocities; LVEF, LV ejection fraction; GLS, global longitudinal strain; cIMT, carotid intima-media thickness; LVH, left ventricle hypertrophy. Values are means ± standard deviation. P-values were calculated using One-way ANOVA tests or Chi-square tests, and Bonferroni correction was used for multiple comparisons*.

# *P < 0.05 vs. NBW group*.

§ *P < 0.05 vs. LBW group*.

**Figure 2:**
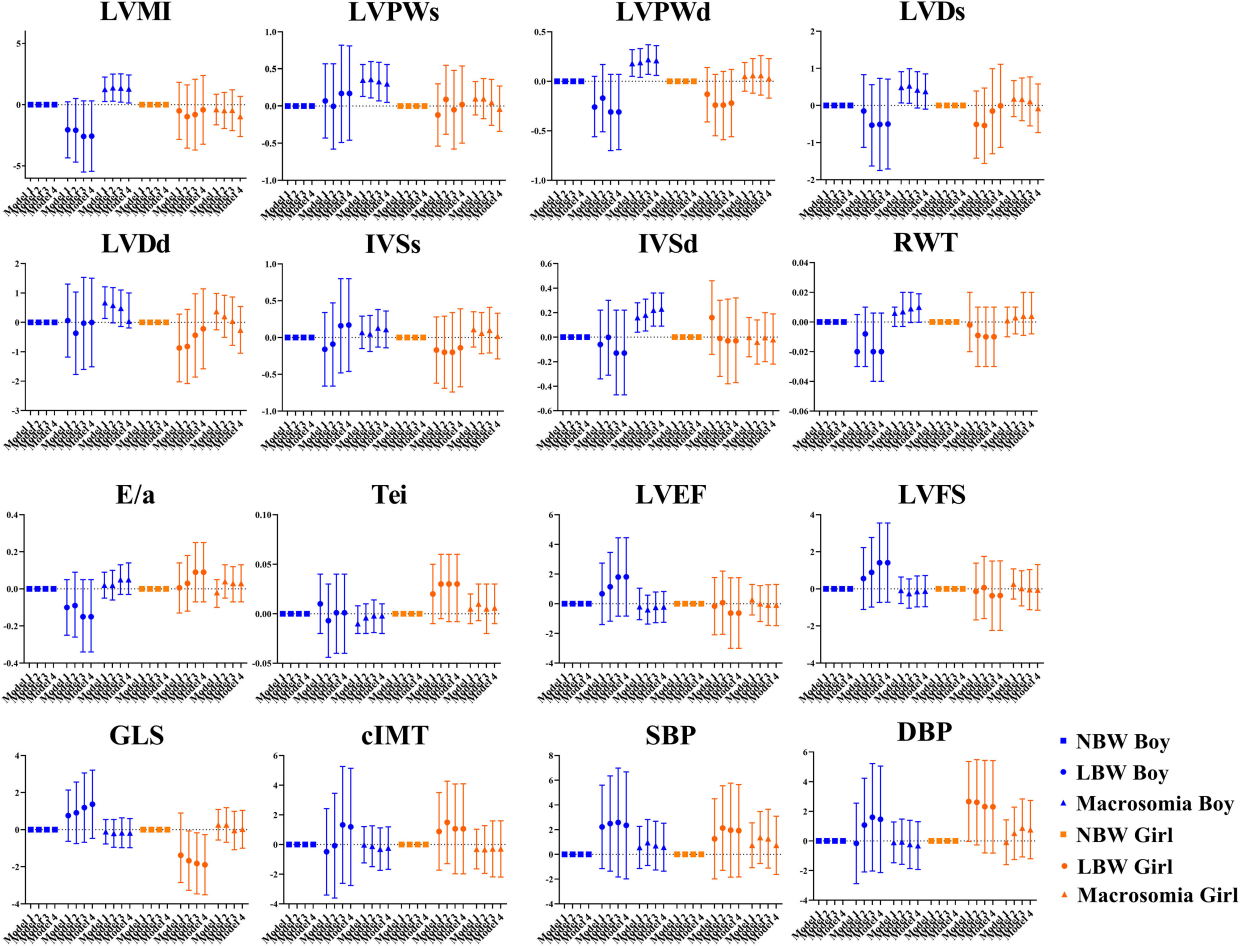
Sex differences in the association between birthweight and cardiovascular parameters at the age of 4 years. Data was presented as mean difference with error bars displaying 95% confidence intervals for multivariable linear regression compared to participants with normal birthweight. Model 1: crude model; Model 2: adjusted for maternal factors including maternal age, ethnicity, educational level, alcohol intake (yes/no) and smoking status (yes/no) during pregnancy, gestational diabetes mellitus (yes/no), and hypertensive disorder during pregnancy (yes/no); Model 3: further adjusted for gestational age; Model 4: further adjusted for current BMI.

The sex-specific association between birthweight and cardiovascular outcome in 4-year-olds is represented in Figure 2. Positive association was found between boys with macrosomia and LVMI (β = 1.29, 95% CI: 0.14, 2.43), LVPWs (β = 0.30, 95% CI: 0.05, 0.56), LVPWd (β = 0.21, 95% CI: 0.06, 0.36), and IVSd (β = 0.23, 95% CI: 0.09, 0.36), while no statistical significance was found in blood pressure, cIMT, or LV function variables. Negative association was found between LBW girls and GLS (β = −1.89, 95% CI: −3.51, −0.27). There was no obvious association found between 4-year-old girls with macrosomia and cardiovascular outcome.

### Sex-Disparity in the Risk of Undesirable Cardiovascular Outcomes in 4-Year-Olds

The risk of LVH in 4-year-old children with macrosomia is shown in Table 3. Children with macrosomia were conferred with a higher risk of LVH compared to NBW group with an odds ratio (OR) of 1.97 (95% CI: 1.01, 3.85) with full adjustment. We further investigated the sex-specific relationship between macrosomia and risk of LVH. Boys with macrosomia were exposed to a higher risk of LVH with OR of 2.79 (95% CI: 1.17, 6.63), indicating that macrosomia was an independent risk factor for LVH among pre-school-aged boys. Based on the results of logistic regression, higher birthweight seemed to have little effect on cardiovascular outcome in 4-year-old girls.

**Table 3:** Odds ratios of LVH in the macrosomia group compared with the NBW group by gender.

**Sex**	**Crude model**		**Adjusted model**	
	**OR (95% CI)**	***P*-value**	**OR (95% CI)**	***P*-value**
**Total**				
NBW	Reference		Reference	
Macrosomia	1.69 (0.98, 2.93)	0.059	**1.97 (1.01, 3.85)**	**0.046**
**Boys**				
NBW	Reference		Reference	
Macrosomia	**2.40 (1.24, 4.66)**	**0.010**	**2.79 (1.17, 6.63)**	**0.020**
**Girls**				
NBW	Reference		Reference	
Macrosomia	0.82 (0.28, 2.40)	0.712	1.17 (0.29, 4.72)	0.824

*LVH, left ventricle hypertrophy; NBW, normal birthweight; OR, odds ratio; CI, confidence interval*.

*Gestational age, current BMI, current SBP, current DBP and maternal factors including maternal age, ethnicity, educational level, alcohol intake (yes/no) and smoking status (yes/no) during pregnancy, gestational diabetes mellitus (yes/no), and hypertensive disorder during pregnancy (yes/no) were further adjusted. Bold values in the table indicate to statistically significant P-value*.

## Discussion

In this study, we demonstrated a significant association between birthweight and cardiovascular parameter changes in pre-school-aged children. Furthermore, for the first time, we found sex-disparity in the association between birthweight and cardiovascular outcome, which developed at an early age of 4 years.

Screening of early cardiovascular changes in children was important as previous studies claimed that adverse LV structure and function changes were important factors influencing cardiac remodeling (27, 28). Myocardial differences that developed with compensation at the expense of cardiac function in childhood could translate into undesirable cardiovascular outcomes in adulthood (29–31). Based on the comprehensive evaluation of cardiac structure and function, our results provided evidence for early detection of cardiac structural and functional changes in children with high risk at pre-school age. Some intervention studies have indicated that there was a reversibility in adverse cardiac adaption at early age, and we would discuss that phenomenon in our further longitudinal study.

In our study, sex-disparity was found to exist in the early adaptation of the cardiovascular system to adverse exposure during childhood. Among boys, the structural changes could develop earlier than functional changes, as cardiac structural changes and increased risk of LVH were observed at an early age of 4 years in children with macrosomia, whereas the cardiac function and peripheral vascular structure presented no obvious changes. However, no obvious structural changes were found in girls. Given the negative association found between LBW and GLS, we hypothesized that girls could develop functional changes earlier, rather than structural changes. Though a changes in GLS alone could not predict decline of cardiac function, it indicated an early-development of impaired contractile mechanics.

Furthermore, we revealed sex-disparity in the association between birthweight and cardiovascular outcome, indicating that there might be different determinants of cardiovascular outcome between boys and girls. The increased risk of LVH was found in boys with macrosomia, while there was no significant result found in girls. This finding was consistent with the results of some studies conducted in adults. Direct association between LVM at adolescence with birthweight (32, 33) and male sex (34) has been reported, which is in line with our results. Previous studies also found that LV structure was determined by somatic growth, and the current weight also played a role (35). Though there was no statistically significant result in girls, a slight upward trend of parameters related to LV geometry was still observed with increased birthweight. Based on the results of our study, we hypothesized that birthweight could be an important determinant in cardiovascular structure during early childhood for boys, and somatic growth and current body size could be more important for girls. This hypothesis needs further validation in a larger cohort study or animal models.

The possible mechanism of sex dimorphism in the increased risk of cardiovascular structural changes in macrosomic children is explained in the following lines. Genetic factors were considered first. The presence of sex chromosomes resulted in the difference between the bodies of males and females at the most fundamental genetic level (36–38). Indeed, some scholars has declared that every cell had its own sex (39). Followed by imprinting from parents, incomplete X-inactivation, and epigenetic modification, the genes also influenced the development of cardiovascular organs (40). Besides, body fat distribution was another important factor. The difference in body fatness and adipose tissue distribution in different genders has been well documented (41). Males were more prone to the deposition of abdominal fat, especially around the abdominal internal organs. A previous study also reported a stronger increment in LVM in male mice when compared to female mice, on exposure to a high-fat diet (42). Furthermore, the sympathetic activation could also play a role. Many studies found that sympathetic activation was much stronger in men, and the sex-specific requirement for α1-adrenergic receptors had been previously reported (43). Although a body of evidence indicated that sex hormone had a large influence on the development of cardiovascular disease (11, 40, 44), we thought that the effect of hormone in this study was little due to the slight difference in sex hormone in pre-school-aged children. Moreover, the social determinant difference such as physical activity should also be taken into consideration.

The investigation of our study was based on the prospective birth cohort study in China, with a detailed assessment of cardiovascular parameters during early childhood, which was seldom performed in previous epidemiology studies. Furthermore, for the first time, our study revealed that sex dimorphism developed in the association between macrosomia and increased risk of cardiovascular disease in early childhood. The limitations in our study should also be considered. Firstly, the risk of undesirable cardiovascular outcomes in children with LBW could not be fully explored due to fewer LBW children and low incidence of LVH among them. A larger sample size is expected for further investigation. Second, the influence of growth trajectories during early life was not considered for investigation in this study. As children with LBW/macrosomia usually presented a catch-up/catch-down growth (45–47), the role of the growth pattern in cardiovascular health among these children deserved more attention. Finally, information regarding physical activity was not included in this study. Physical activity was a risk factor for cardiovascular disease across the lifespan. The gender difference in adoption of health behaviors in early life has been illustrated (48), and it was observed that boys were encouraged for the greater pursuit of physical activity even from infancy. The gender difference and effect of physical activity need more consideration.

In conclusion, early adaptation of the cardiovascular system with structural changes was found in children with LBW or macrosomia. For the first time, our study revealed the sex-disparity in the association between birthweight and cardiovascular parameters, where boys with macrosomia had heavier and thicker LV and were at a higher risk of LVH. Results of our work led to a better understanding of the sex-disparity in cardiovascular disease, providing evidence for more precise and effective interventions to reduce the risk of undesirable cardiovascular outcomes in children with risk factors of cardiovascular diseases.

## Data Availability Statement

The raw data supporting the conclusions of this article will be made available by the authors, without undue reservation.

## Ethics Statement

The studies involving human participants were reviewed and approved by Ethical Committee of Xinhua Hospital Affiliated to Shanghai Jiao Tong University School of Medicine. Written informed consent to participate in this study was provided by the participants' legal guardian/next of kin.

## Author Contributions

KS, SC, and JW conceived and designed the study and collected data. HW and BD prepared an analytical plan, analyzed data, and drafted the initial manuscript. YW, YN, and ZL were involved in echocardiography examinations and data collection. FO collaborated in the revision and interpretation of the data and results. All authors reviewed and revised the manuscript, approved the final manuscript as submitted, and agreed to be accountable for all aspects of the work.

## Funding

This work was supported by the National Key R&D Program of China (grant nos: 2018YFC1002400 to 403), Collaborative Innovation Program of Shanghai Municipal Health Commission (grant no: 2020CXJQ01), and Hospital Funded Clinical Research, Clinical Research Unit, Xinhua Hospital Affiliated to Shanghai Jiao Tong University School of Medicine (grant no: 19XHCR06B).

## Conflict of Interest

The authors declare that the research was conducted in the absence of any commercial or financial relationships that could be construed as a potential conflict of interest.

## Publisher's Note

All claims expressed in this article are solely those of the authors and do not necessarily represent those of their affiliated organizations, or those of the publisher, the editors and the reviewers. Any product that may be evaluated in this article, or claim that may be made by its manufacturer, is not guaranteed or endorsed by the publisher.

## Abbreviations

SBC: Shanghai Birth Cohort
LBW: low birthweight
NBW: normal birthweight
SBP: systolic blood pressure
DBP: diastolic blood pressure
BMI: body mass index
LV: left ventricle
LVH: left ventricle hypertrophy
LVMI: LV mass indexed to the height in m^2.7^
LVPWs: LV posterior wall thickness in systole
LVPWd: LV posterior wall thickness in diastole
LVDs: LV internal diameter in systole
LVDd: LV internal diameter in diastole
IVSs: interventricular septum thickness in systole
IVSd: interventricular septum thickness in diastole
RWT: relative wall thickness
LVEF: LV ejection fraction
GLS: global longitudinal strain
cIMT: carotid intima-media thickness.
