# Supplementary material for: Sex-Disparity in the Association Between Birthweight and Cardiovascular Parameters in 4-Year-Old Children: A Chinese Cohort Study
Source: Front Nutr. 2021 Oct 26;8:756512. doi: 10.3389/fnut.2021.756512 (PMC8576373; doi:10.3389/fnut.2021.756512)
Supplement: Supplementary file 1 [file Data_Sheet_1.docx]

***Supplementary Material***

**Supplementary Table 1.** The association between birthweight and cardiovascular parameters at the age of 4. LBW: low birthweight; NBW: normal birthweight; LV: left ventricle; LVMI, LV mass indexed to the height in m2.7; LVPWs, LV posterior wall thicknesses in systole; LVPWd, LV posterior wall thicknesses in diastole; LVDs, LV internal diameter in systole; LVDd, LV internal diameter in diastole; IVSs, interventricular septum thickness in systole; IVSd, interventricular septum thickness in diastole; RWT, relative wall thickness; E, mitral early wave velocities; a, mitral late wave velocities; LVEF, LV ejection fraction; LVFS, LV fraction shortening; GLS, global longitudinal strain; cIMT, carotid intima-media thickness; SBP: systolic blood pressure; DBP: diastolic blood pressure. Data was presented as mean difference with 95% confidence intervals for multivariable linear regression compared to participants with normal birthweight. Model 1: crude model; Model 2: adjusted for maternal factors including maternal age, ethnicity, educational level, alcohol intake (yes/no) and smoking status (yes/no) during pregnancy, gestational diabetes mellitus (yes/no) and hypertensive disorder of pregnancy (yes/no); Model 3: further adjusted for gestational age; Model 4: further adjusted for current BMI.

**Supplementary Table 2.** Sex differences in the association between birthweight and cardiovascular parameters at the age of 4. A: The association between birthweight and cardiovascular parameters in 4-year-old boys; B. The association between birthweight and cardiovascular parameters in 4-year-old girls. LBW: low birthweight; NBW: normal birthweight; LV: left ventricle; LVMI, LV mass indexed to the height in m2.7; LVPWs, LV posterior wall thicknesses in systole; LVPWd, LV posterior wall thicknesses in diastole; LVDs, LV internal diameter in systole; LVDd, LV internal diameter in diastole; IVSs, interventricular septum thickness in systole; IVSd, interventricular septum thickness in diastole; RWT, relative wall thickness; E, mitral early wave velocities; a, mitral late wave velocities; LVEF, LV ejection fraction; LVFS, LV fraction shortening; GLS, global longitudinal strain; cIMT, carotid intima-media thickness; SBP: systolic blood pressure; DBP: diastolic blood pressure. Data was presented as mean difference with 95% confidence intervals for multivariable linear regression compared to participants with normal birthweight. Model 1: crude model; Model 2: adjusted for maternal factors including maternal age, ethnicity, educational level, alcohol intake (yes/no) and smoking status (yes/no) during pregnancy, gestational diabetes mellitus (yes/no) and hypertensive disorder of pregnancy (yes/no); Model 3: further adjusted for gestational age; Model 4: further adjusted for current BMI.

Supplementary Table 1. The association between birthweight and cardiovascular parameters at the age of 4

|  | Model 1 | | | | | Model 2 | | | | | Model 3 | | | | | Model 4 | | | | |
| --- | --- | --- | --- | --- | --- | --- | --- | --- | --- | --- | --- | --- | --- | --- | --- | --- | --- | --- | --- | --- |
|  | NBW | LBW | | Macrosomia | | NBW | LBW | | Macrosomia | | NBW | LBW | | Macrosomia | | NBW | LBW | | Macrosomia | |
|  |  | β (95% CI) | P-value | β (95% CI) | P-value |  | β (95% CI) | P-value | β (95% CI) | P-value |  | β (95% CI) | P-value | β (95% CI) | P-value |  | β (95% CI) | P-value | β (95% CI) | P-value |
| LVMI | reference | -1.28 (-2.93, 0.37) | -0.129 | 0.63 (-0.17, 1.42) | 0.123 | reference | -1.56 (-3.41, 0.29) | 0.100 | 0.96 (0.05, 1.86) | 0.038 | reference | -1.62 (-3.67, 0.44) | 0.123 | 0.98 (0.02, 1.95) | 0.046 | reference | -1.49 (-3.51,0.53) | 0.147 | 0.74 (-0.22, 1.69) | 0.129 |
| LVPWs | reference | -0.05 (-0.38, 0.28) | 0.767 | 0.25 (0.10, 0.41) | 0.002 | reference | -0.01 (-0.38, 0.37) | 0.978 | 0.31 (0.13, 0.49) | 0.001 | reference | -0.01 (-0.43, 0.40) | 0.947 | 0.31 (0.11, 0.50) | 0.002 | reference | 0.01 (-0.40, 0.42) | 0.967 | 0.26 (0.06, 0.45) | 0.009 |
| LVPWd | reference | -0.20 (-0.41, 0.01) | 0.058 | 0.13 (0.03, 0.23) | 0.009 | reference | -0.23 (-0.46, 0.002) | 0.053 | 0.17 (0.06, 0.28) | 0.003 | reference | -0.29 (-0.55, -0.03) | 0.027 | 0.19 (0.07, 0.31） | 0.002 | reference | -0.28 (-0.54, -0.03) | 0.03 | 0.18 (0.06, 0.30) | 0.003 |
| LVDs | reference | -0.39 (-1.07, 0.29) | 0.263 | 0.42 (0.10, 0.75) | 0.011 | reference | -0.54 (-1.31, 0.22) | 0.161 | 0.45 (0.08, 0.82) | 0.017 | reference | -0.30 (-1.15, 0.56) | 0.492 | 0.39 (-0.01, 0.80) | 0.053 | reference | -0.24 (-1.07, 0.59) | 0.571 | 0.27 (-0.12, 0.66) | 0.179 |
| LVDd | reference | -0.52 (-1.41, 0.36) | 0.244 | 0.63 (0.20, 1.05) | 0.004 | reference | -0.66 (-1.64, 0.32) | 0.185 | 0.58 (0.10, 1.05) | 0.018 | reference | -0.31 (-1.40, 0.78) | 0.574 | 0.52 (0.01, 1.03) | 0.047 | reference | -0.22 (-1.27, 0.82) | 0.677 | 0.33 (-0.17, 0.82) | 0.194 |
| IVSs | reference | -0.19(-0.53, 0.15) | 0.274 | 0.11 (-0.06, 0.27) | 0.200 | reference | -0.18 (-0.56, 0.20) | 0.346 | 0.13 (-0.05, 0.32) | 0.167 | reference | -0.10 (-0.53, 0.32) | 0.639 | 0.22 (0.02, 0.42) | 0.032 | reference | -0.08 (-0.50, 0.34) | 0.707 | 0.17 (-0.02, 0.37) | 0.086 |
| IVSd | reference | 0.06 (-0.14, 0.26) | 0.562 | 0.09 (-0.01, 0.19) | 0.060 | reference | -0.03 (-0.25, 0.19) | 0.813 | 0.13 (0.02, 0.23) | 0.020 | reference | -0.10 (-0.34, 0.15) | 0.432 | 0.16 (0.05, 0.28) | 0.004 | reference | -0.10, (-0.34, 0.15) | 0.441 | 0.16 (0.05, 0.28) | 0.006 |
| RWT | reference | -0.10 (-0.02, 0.01) | 0.241 | 0.004 (-0.003,0.01) | 0.268 | reference | -0.10 (-0.02, 0.01) | 0.208 | 0.01 (-0.001,0.01) | 0.084 | reference | -0.02 (-0.03, 0.001) | 0.059 | 0.01 (0.001, 0.02) | 0.038 | reference | -0.02 (-0.03, 0.001) | 0.054 | 0.01 (0.001, 0.02) | 0.025 |
| E/a | reference | -0.03 (-0.13, 0.07) | 0.604 | -0.002 (-0.06, 0.05) | 0.931 | reference | -0.001 (-0.11, 0.11) | 0.979 | 0.03 (-0.03, 0.09) | 0.315 | reference | 0.01 (-0.12, 0.13) | 0.902 | 0.03 (-0.03, 0.09) | 0.328 | reference | 0.001 (-0.12, 0.12) | 0.986 | 0.04 (-0.02, 0.10) | 0.232 |
| Tei | reference | 0.02 (-0.01, 0.04) | 0.145 | -0.001 (-0.01, 0.01) | 0.920 | reference | 0.01 (-0.01, 0.04) | 0.289 | 0.003 (-0.01, 0.02) | 0.571 | reference | 0.02 (-0.01, 0.04) | 0.186 | 0.002 (-0.01, 0.02) | 0.704 | reference | 0.02 (-0.01, 0.04) | 0.186 | 0.002 (-0.01, 0.02) | 0.716 |
| LVEF | reference | 0.21 (-1.20, 1.61) | 0.773 | -0.004 (-0.68, 0.67) | 0.991 | reference | 0.52 (-1.05, 2.08) | 0.517 | -0.12 (-0.88, 0.64) | 0.755 | reference | 0.33 (-1.42, 2.09) | 0.709 | -0.02 (-0.84, 0.80) | 0.959 | reference | 0.31 (-1.44, 2.07) | 0.725 | 0.02 (-0.81, 0.84) | 0.966 |
| LVFS | reference | 0.17 (-0.96, 1.29) | 0.773 | 0.07 (-0.47, 0.61) | 0.801 | reference | 0.38 (-0.87, 1.63) | 0.551 | -0.05 (-0.66, 0.56) | 0.874 | reference | 0.28 (-1.12, 1.68) | 0.696 | 0.03 (-0.63, 0.68) | 0.940 | reference | 0.27 (-1.13, 1.68) | 0.703 | 0.04 (-0.62, 0.70) | 0.905 |
| GLS | reference | -0.27 (-1.28, 0.74) | 0.603 | -0.003 (-0.52, 0.52) | 0.991 | reference | -0.33 (-1.48, 0.82) | 0.574 | -0.06 (-0.64, 0.52) | 0.832 | reference | -0.37 (-1.58, 0.85) | 0.555 | -0.13 (-0.75, 0.49) | 0.674 | reference | --0.30 (-1.50, 0.90) | 0.622 | -0.10 (-0.72, 0.51) | 0.740 |
| cIMT | reference | 0.22 (-1.73, 2.16) | 0.828 | -0.12 (-1.02, 0.78) | 0.793 | reference | 0.71 (-1.46, 2.88) | 0.522 | -0.23 (-1.25, 0.79) | 0.661 | reference | 1.25 (-1.12, 3.63) | 0.300 | -0.16 (-1.26, 0.94) | 0.777 | reference | 1.18 (-1.19, 3.55) | 0.328 | -0.10 (-1.20, 1.01) | 0.866 |
| SBP | reference | 1.78 (-0.60, 4.15) | 0.142 | 0.91 (-0.36, 2.17) | 0.159 | reference | 2.19 (-0.40, 4.77) | 0.097 | 1.51 (0.11, 2.91) | 0.034 | reference | 2.01 (-0.89, 4.92) | 0.174 | 1.44 (-0.08, 2.95) | 0.063 | reference | 1.89 (-0.96, 4.73) | 0.193 | 1.10 (-0.39, 2.58) | 0.148 |
| DBP | reference | 1.21 (-0.71, 3.13) | 0.215 | -0.10 (-1.12, 0.92) | 0.850 | reference | 1.95 (-0.17, 4.07) | 0.070 | 0.08 (-1.06, 1.23) | 0.888 | reference | 1.98 (-0.38, 4.35) | 0.101 | 0.16 (-1.07, 1.40) | 0.794 | reference | 1.93 (-0.42, 4.29) | 0.108 | 0.04 (-1.20, 1.27) | 0.995 |

Supplementary Table 2A. The association between birthweight and cardiovascular parameters in 4-year-old boys

|  | Model 1 | | | | | Model 2 | | | | | Model 3 | | | | | Model 4 | | | | |
| --- | --- | --- | --- | --- | --- | --- | --- | --- | --- | --- | --- | --- | --- | --- | --- | --- | --- | --- | --- | --- |
|  | NBW | LBW | | Macrosomia | | NBW | LBW | | Macrosomia | | NBW | LBW | | Macrosomia | | NBW | LBW | | Macrosomia | |
|  |  | β (95% CI) | P-value | β (95% CI) | P-value |  | β (95% CI) | P-value | β (95% CI) | P-value |  | β (95% CI) | P-value | β (95% CI) | P-value |  | β (95% CI) | P-value | β (95% CI) | P-value |
| LVMI | reference | -2.05 (-4.34, 0.24) | 0.08 | 1.26(0.26, 2.26) | 0.014 | reference | -2.09 (-4.69, 0.51) | 0.114 | 1.39 (0.28, 2.50) | 0.014 | reference | -2.59 (-5.50, 0.31) | 0.080 | 1.36 (0.21, 2.52) | 0.021 | reference | -2.57 (-5.44, 0.31) | 0.080 | 1.29 (0.14, 2.43) | 0.028 |
| LVPWs | reference | 0.07 (-0.43, 0.57) | 0.773 | 0.35 (0.13, 0.56) | 0.002 | reference | -0.003 (-0.58, 0.57) | 0.993 | 0.36 (0.11, 0.60) | 0.005 | reference | 0.17 (-0.49, 0.82) | 0.617 | 0.33 (0.07, 0.59) | 0.013 | reference | 0.17 (-0.46, 0.81) | 0.590 | 0.30 (0.05, 0.56) | 0.018 |
| LVPWd | reference | -0.26 (-0.56, 0.05) | 0.099 | 0.18 (0.05, 0.32) | 0.007 | reference | -0.17 (-0.51, 0.17) | 0.330 | 0.19 (0.04, 0.33) | 0.011 | reference | -0.31 (-0.70, 0.07) | 0.108 | 0.22 (-0.07, 0.37) | 0.005 | reference | -0.31 (-0.69, 0.07) | 0.109 | 0.21 (0.06, 0.36) | 0.006 |
| LVDs | reference | -0.15 (-1.13, 0.83) | 0.765 | 0.49 (0.07, 0.91) | 0.023 | reference | -0.53 (-1.63, 0.56) | 0.340 | 0.53 (0.06, 0.99) | 0.027 | reference | -0.51 (-1.75, 0.73) | 0.416 | 0.42 (-0.07, 0.91) | 0.091 | reference | -0.50 (-1.71, 0.71) | 0.419 | 0.38 (-0.10, 0.85) | 0.124 |
| LVDd | reference | 0.06 (-1.18, 1.30) | 0.925 | 0.67 (0.13, 1.21) | 0.015 | reference | -0.37 (-1.77, 1.03) | 0.606 | 0.58 (-0.02, 1.18) | 0.057 | reference | -0.03 (-1.60, 1.54) | 0.968 | 0.48 (-0.14, 1.10) | 0.130 | reference | -0.004 (-1.51, 1.50) | 0.996 | 0.40 (-0.19, 1.00) | 0.183 |
| IVSs | reference | -0.16 (-0.66, 0.34) | 0.535 | 0.07 (-0.15, 0.29) | 0.546 | reference | -0.09 (-0.66, 0.47) | 0.744 | 0.05 (-0.19, 0.30) | 0.656 | reference | 0.16 (-0.48, 0.80) | 0.618 | 0.13 (-0.13, 0.38) | 0.327 | reference | 0.17 (-0.46, 0.80) | 0.597 | 0.11 (-0.14, 0.36) | 0.399 |
| IVSd | reference | -0.06 (-0.34, 0.22) | 0.686 | 0.16 (0.04, 0.28) | 0.009 | reference | -0.001 (-0.31, 0.30) | 0.993 | 0.18 (0.05, 0.31) | 0.006 | reference | -0.13 (-0.47, 0.22) | 0.468 | 0.22 (0.09, 0.36) | 0.001 | reference | -0.13 (-0.47, 0.22) | 0.468 | 0.23 (0.09, 0.36) | 0.001 |
| RWT | reference | -0.02 (-0.03, 0.004) | 0.134 | 0.006 (-0.003, 0.01) | 0.187 | reference | -0.01 (-0.03, 0.01) | 0.499 | 0.01 (-0.003, 0.02) | 0.156 | reference | -0.02 (-0.04, 0.01) | 0.131 | 0.01 (-0.001, 0.02) | 0.060 | reference | -0.02 (-0.04, 0.01) | 0.129 | 0.01 (-0.001, 0.02) | 0.055 |
| E/a | reference | -0.10 (-0.25, 0.05) | 0.205 | 0.02 (-0.05, 0.09) | 0.560 | reference | -0.09 (-0.26, 0.09) | 0.330 | 0.02 (-0.06, 0.10) | 0.577 | reference | -0.15 (-0.34, 0.05) | 0.138 | 0.05 (-0.03, 0.13) | 0.249 | reference | -0.15 (-0.34, 0.05) | 0.138 | 0.05 (-0.03, 0.14) | 0.223 |
| Tei | reference | 0.01 (-0.02, 0.04) | 0.576 | -0.01 (-0.02, 0.01) | 0.399 | reference | -0.01 (-0.04, 0.03) | 0.695 | -0.004 (-0.02, 0.01) | 0.611 | reference | 0.001 (-0.04, 0.04) | 0.949 | -0.002 (-0.02, 0.01) | 0.801 | reference | 0.001 (-0.04, 0.04) | 0.951 | -0.002 (-0.02, 0.01) | 0.809 |
| LVEF | reference | 0.67 (-1.40, 2.74) | 0.524 | -0.20 (-1.08, 0.69) | 0.665 | reference | 1.14 (-1.18, 3.46) | 0.335 | -0.40 (-1.37, 0.58) | 0.427 | reference | 1.80 (-0.84, 4.44) | 0.180 | -0.25 (-1.28, 0.79) | 0.646 | reference | 1.81 (-0.83, 4.45) | 0.178 | -0.21 (-1.25, 0.82) | 0.688 |
| LVFS | reference | 0.56 (-1.11, 2.23) | 0.510 | -0.08(-0.79, 0.64) | 0.834 | reference | 0.89 (-0.98, 2.77) | 0.350 | -0.25 (-1.04, 0.54) | 0.535 | reference | 1.41 (-0.73, 3.55) | 0.195 | -0.14 (-0.97,0.70) | 0.750 | reference | 1.41 (-0.72, 3.55) | 0.195 | -0.12 (-0.96, 0.72) | 0.777 |
| GLS | reference | 0.76 (-0.63, 2.15) | 0.286 | -0.11 (-0.77, 0.55) | 0.753 | reference | 0.91 (-0.75, 2.57) | 0.282 | -0.20 (-0.95, 0.55) | 0.603 | reference | 1.19 (-0.68, 3.06) | 0.212 | -0.17 (-0.97, 0.64) | 0.684 | reference | 1.37 (-0.47, 3.21) | 0.143 | -0.19 (-0.98, 0.60) | 0.629 |
| cIMT | reference | -0.49 (-3.41, 2.42) | 0.740 | -0.02 (-1.24, 1.21) | 0.979 | reference | -0.08 (-3.61, 3.45) | 0.966 | -0.12 (-1.50, 1.25) | 0.862 | reference | 1.32 (-2.62, 5.27) | 0.510 | -0.31 (-1.74, 1.12) | 0.670 | reference | 1.19 (-2.76, 5.14) | 0.554 | -0.24 (-1.67, 1.19) | 0.740 |
| SBP | reference | 2.22 (-1.15, 5.60) | 0.196 | 0.57 (-1.14, 2.28) | 0.513 | reference | 2.49 (-1.36, 6.35) | 0.204 | 0.96 (-0.90, 2.82) | 0.309 | reference | 2.58 (-1.82, 6.98) | 0.250 | 0.71 (-1.26, 2.68) | 0.479 | reference | 2.35 (-1.98, 6.67) | 0.288 | 0.58 (-1.36, 2.52) | 0.559 |
| DBP | reference | -0.16 (-2.88, 2.55) | 0.906 | -0.10 (-1.47, 1.28) | 0.891 | reference | 1.07 (-2.09, 4.23) | 0.506 | -0.06 (-1.58, 1.47) | 0.943 | reference | 1.60 (-2.03,5.22) | 0.387 | -0.23 (-1.86, 1.39) | 0.778 | reference | 1.46 (-2.13, 5.05) | 0.425 | -0.31 (-1.92, 1.30) | 0.705 |

Supplementary Table 2B. The association between birthweight and cardiovascular parameters in 4-year-old girls

|  | Model 1 | | | | | Model 2 | | | | | Model 3 | | | | | Model 4 | | | | |
| --- | --- | --- | --- | --- | --- | --- | --- | --- | --- | --- | --- | --- | --- | --- | --- | --- | --- | --- | --- | --- |
|  | NBW | LBW | | Macrosomia | | NBW | LBW | | Macrosomia | | NBW | LBW | | Macrosomia | | NBW | LBW | | Macrosomia | |
|  |  | β (95% CI) | P-value | β (95% CI) | P-value |  | β (95% CI) | P-value | β (95% CI) | P-value |  | β (95% CI) | P-value | β (95% CI) | P-value |  | β (95% CI) | P-value | β (95% CI) | P-value |
| LVMI | reference | -0.50 (-2.83, 1.83) | 0.672 | -0.39 (-1.64, 0.85) | 0.537 | reference | -0.97 (-3.55, 1.62) | 0.465 | -0.47 (-1.94, 1.00) | 0.533 | reference | -0.81 (-3.70, 2.08) | 0.581 | -0.45 (-2.12, 1.22) | 0.599 | reference | -0.42 (-3.23, 2.39) | 0.769 | -0.96 (-2.60, 0.68) | 0.251 |
| LVPWs | reference | -0.12 (-0.54, 0.30) | 0.571 | 0.10 (-0.12, 0.33) | 0.360 | reference | 0.09 (-0.38, 0.55) | 0.720 | 0.10 (-0.17, 0.37) | 0.469 | reference | -0.05 (-0.58, 0.48) | 0.864 | 0.05 (-0.26, 0.36) | 0.753 | reference | 0.02 (-0.50, 0.54) | 0.944 | -0.04 (-0.34, 0.27) | 0.814 |
| LVPWd | reference | -0.13 (-0.41, 0.14) | 0.342 | 0.05 (-0.10, 0.19) | 0.531 | reference | -0.24 (-0.55, 0.07) | 0.123 | 0.06 (-0.12, 0.23) | 0.523 | reference | -0.24 (-0.59, 0.10) | 0.166 | 0.06 (-0.14, 0.26) | 0.564 | reference | -0.22 (-0.56, 0.12) | 0.205 | 0.03 (-0.17, 0.23) | 0.764 |
| LVDs | reference | -0.51(-1.42, 0.39) | 0.266 | 0.18 (-0.30, 0.67) | 0.461 | reference | -0.54 (-1.56, 0.47) | 0.296 | 0.17 (-0.41, 0.74) | 0.572 | reference | -0.15 (-1.30, 0.99) | 0.794 | 0.11 (-0.55, 0.77) | 0.743 | reference | -0.01 (-1.13, 1.11) | 0.986 | -0.08 (-0.73, 0.58) | 0.817 |
| LVDd | reference | -0.87 (-2.02, 0.28) | 0.139 | 0.36 (-0.25, 0.98) | 0.246 | reference | -0.82 (-2.08, 0.44) | 0.202 | 0.20 (-0.51, 0.92) | 0.579 | reference | -0.44 (-1.86, 0.97) | 0.539 | 0.04 (-0.78, 0.86) | 0.922 | reference | -0.22 (-1.58, 1.14) | 0.754 | -0.26 (-1.05, 0.54) | 0.527 |
| IVSs | reference | -0.17 (-0.62, 0.28) | 0.458 | 0.11 (-0.13, 0.35) | 0.354 | reference | -0.20 (-0.69, 0.29) | 0.425 | 0.06 (-0.22, 0.34) | 0.655 | reference | -0.20 (-0.74, 0.34) | 0.468 | 0.10 (-0.21 0.41) | 0.537 | reference | -0.14 (-0.67, 0.39) | 0.608 | 0.02 (-0.29, 0.33) | 0.910 |
| IVSd | reference | 0.16 (-0.14, 0.46) | 0.293 | -0.001 (-0.16, 0.16) | 0.991 | reference | -0.01 (-0.32, 0.30) | 0.942 | -0.04 (-0.22, 0.14) | 0.664 | reference | -0.03 (-0.38, 0.31) | 0.846 | -0.003 (-0.20, 0.20) | 0.976 | reference | -0.03 (-0.37, 0.32) | 0.887 | -0.02 (-0.22, 0.19) | 0.882 |
| RWT | reference | -0.002 (-0.02, 0.02) | 0.829 | 0.001 (-0.01, 0.01) | 0.833 | reference | -0.01 (-0.03, 0.01) | 0.351 | 0.003 (-0.01, 0.01) | 0.645 | reference | -0.01 (-0.03, 0.01) | 0.285 | 0.004 (-0.01, 0.02) | 0.511 | reference | -0.01 (-0.03, 0.01) | 0.274 | 0.005 (-0.01, 0.02) | 0.474 |
| E/a | reference | 0.01 (-0.13, 0.14) | 0.930 | -0.02 (-0.10, 0.05) | 0.542 | reference | 0.03 (-0.12, 0.18) | 0.680 | 0.04 (-0.05, 0.13) | 0.328 | reference | 0.09 (-0.07, 0.25) | 0.251 | 0.03 (-0.07, 0.12) | 0.618 | reference | 0.09 (-0.07, 0.25) | 0.272 | 0.03 (-0.07, 0.13) | 0.519 |
| Tei | reference | 0.02 (-0.01, 0.05) | 0.128 | 0.01 (-0.01, 0.02) | 0.516 | reference | 0.03 (-0.01, 0.06) | 0.097 | 0.01 (-0.01, 0.03) | 0.233 | reference | 0.03 (-0.01, 0.06) | 0.137 | 0.01 (-0.02, 0.03) | 0.635 | reference | 0.03 (-0.01, 0.06) | 0.142 | 0.01(-0.01, 0.03) | 0.567 |
| LVEF | reference | -0.16 (-2.09, 1.77) | 0.870 | 0.27 (-0.76, 1.30) | 0.613 | reference | 0.07 (-2.06, 2.20) | 0.949 | 0.01 (-1.20, 1.22) | 0.982 | reference | -0.63 (-3.01, 1.75) | 0.605 | -0.09 (-1.46, 1.28) | 0.897 | reference | -0.63 (-3.01, 1.76) | 0.606 | -0.09 (-1.48, 1.30) | 0.897 |
| LVFS | reference | -0.14 (-1.67, 1.39) | 0.857 | 0.26 (-0.55, 1.08) | 0.526 | reference | 0.07 (-1.60, 1.75) | 0.933 | 0.03 (-0.92, 0.98) | 0.950 | reference | -0.37 (-2.24, 1.50) | 0.695 | -0.04 (-1.12, 1.04) | 0.942 | reference | -0.36 (-2.24, 1.51) | 0.703 | -0.06 (-1.15, 1.04) | 0.916 |
| GLS | reference | -1.38 (-2.85, 0.90) | 0.066 | 0.26 (-0.56, 1.09) | 0.531 | reference | -1.67 (-3.27, -0.07) | 0.041 | 0.26 (-0.68, 1.19) | 0.588 | reference | -1.82 (-3.46, -0.17) | 0.031 | -0.04 (-1.08, 0.99) | 0.934 | reference | -1.89 (-3.51, -0.27) | 0.023 | 0.03 (-1.00, 1.05) | 0.962 |
| cIMT | reference | 0.88 (-1.73, 3.50) | 0.507 | -0.31 (-1.64, 1.03) | 0.654 | reference | 1.49 (-1.29, 4.27) | 0.291 | -0.34 (-1.95, 1.27) | 0.681 | reference | 1.06 (-1.97, 4.08) | 0.492 | -0.29 (-2.18, 1.59) | 0.759 | reference | 1.06 (-1.98, 4.09) | 0.494 | -0.29 (-2.19, 1.60) | 0.761 |
| SBP | reference | 1.26 (-1.98, 4.50) | 0.446 | 0.74 (-1.07, 2.55) | 0.421 | reference | 2.13 (-1.29, 5.55) | 0.222 | 1.37 (-0.73, 3.47) | 0.201 | reference | 1.96 (-1.84, 5.76) | 0.312 | 1.27 (-1.11, 3.65) | 0.296 | reference | 1.92 (-1.82, 5.65) | 0.313 | 0.74 (-1.62, 3.09) | 0.539 |
| DBP | reference | 2.66 (-0.04, 5.36) | 0.054 | -0.09 (-1.60, 1.42) | 0.907 | reference | 2.61 (-0.27, 5.48) | 0.075 | 0.52 (-1.25, 2.28) | 0.564 | reference | 2.31 (-0.81, 5.43) | 0.146 | 0.87 (-1.08, 2.83) | 0.380 | reference | 2.30 (-0.82, 5.42) | 0.148 | 0.76 (-1.20, 2.73) | 0.447 |
